# Spatio-temporal feature learning with reservoir computing for T-cell segmentation in live-cell $\hbox {Ca}^{2+}$ fluorescence microscopy

**DOI:** 10.1038/s41598-021-87607-y

**Published:** 2021-04-15

**Authors:** Fatemeh Hadaeghi, Björn-Philipp Diercks, Daniel Schetelig, Fabrizio Damicelli, Insa M. A. Wolf, René Werner

**Affiliations:** 1 Department of Computational Neuroscience, Center for Experimental Medicine, University Medical Center Hamburg-Eppendorf (UKE), Martinistrasse 52, 20246 Hamburg, Germany; 2 Center for Biomedical Artificial Intelligence (bAIome), University Medical Center Hamburg-Eppendorf (UKE), Martinistrasse 52, 20246 Hamburg, Germany; 3 Department of Biochemistry and Molecular Cell Biology, Center for Experimental Medicine, University Medical Center Hamburg-Eppendorf (UKE), Martinistrasse 52, 20246 Hamburg, Germany

**Keywords:** Biochemistry, Computational biology and bioinformatics, Medical research, Engineering, Mathematics and computing

## Abstract

Advances in high-resolution live-cell $\hbox {Ca}^{2+}$ imaging enabled subcellular localization of early $\hbox {Ca}^{2+}$ signaling events in T-cells and paved the way to investigate the interplay between receptors and potential target channels in $\hbox {Ca}^{2+}$ release events. The huge amount of acquired data requires efficient, ideally automated image processing pipelines, with cell localization/segmentation as central tasks. Automated segmentation in live-cell cytosolic $\hbox {Ca}^{2+}$ imaging data is, however, challenging due to temporal image intensity fluctuations, low signal-to-noise ratio, and photo-bleaching. Here, we propose a reservoir computing (RC) framework for efficient and temporally consistent segmentation. Experiments were conducted with Jurkat T-cells and anti-CD3 coated beads used for T-cell activation. We compared the RC performance with a standard U-Net and a convolutional long short-term memory (LSTM) model. The RC-based models (1) perform on par in terms of segmentation accuracy with the deep learning models for cell-only segmentation, but show improved temporal segmentation consistency compared to the U-Net; (2) outperform the U-Net for two-emission wavelengths image segmentation and differentiation of T-cells and beads; and (3) perform on par with the convolutional LSTM for single-emission wavelength T-cell/bead segmentation and differentiation. In turn, RC models contain only a fraction of the parameters of the baseline models and reduce the training time considerably.

## Introduction

Regulation of cytosolic and organelle $\hbox {Ca}^{2+}$ concentration and initial transient highly localized $\hbox {Ca}^{2+}$ signals ($\hbox {Ca}^{2+}$ microdomains) are essential for T-cell activation and initiation of effective immune responses^1–5^. While mechanistic details of the initial intra-cellular $\hbox {Ca}^{2+}$ elevation and propagation of $\hbox {Ca}^{2+}$ microdomains during T-cell activation remain poorly understood, advances in fluorescence microscopy enabled monitoring subcellular structures and early signaling events throughout T-cell activation with finer spatial and temporal resolution^1, 4, 6^.

With frame rates higher than 40 Hz^4^, a spatial resolution in the order of the diffraction limit and finer, and acquisition periods of several seconds to minutes, in-depth analysis of the imaging data requires efficient, ideally automated post-processing pipelines^7^. A central pipeline building block in live-cell imaging and $\hbox {Ca}^{2+}$ microdomain analysis is the localization and segmentation of cells. Automated cell segmentation in high-resolution live-cell $\hbox {Ca}^{2+}$ imaging data is, however, challenging due to an intrinsically low signal-to-noise ratio, fast $\hbox {Ca}^{2+}$ signaling-based intensity fluctuations, overlaid by intensity changes on longer time-scales, due to, e.g., T-cell activation and photo-bleaching. Depending on the experimental setup, the cells further exhibit motion and deformation^8^. Moreover, if antibody-coated beads are used to mimic cell-cell interaction and to activate the cells^4, 9^, new objects with potentially similar intensity values and appearance than the cells enter the scene.

In this context, the present work describes computationally efficient segmentation approaches tailored to the requirements of live-cell $\hbox {Ca}^{2+}$ microscopy and $\hbox {Ca}^{2+}$ signaling analysis in T-cells. Methodically, the algorithms rely on the principles of reservoir computing (RC)^10^, which builds on the idea of recurrent neural networks (RNNs) to extract spatio-temporal features to achieve temporally consistent data analysis results and provides a computationally efficient model training and light-weight models in comparison to deep learning-based RNNs.

### Related work

Image post-processing workflows for $\hbox {Ca}^{2+}$ microscopy data commonly provide (semi-)automatic solutions to problems directly associated with the imaging process, such as bleaching correction, deconvolution, and emission-channel alignment in dual-wavelength measurements^4, 7, 11–13^. Cell segmentation is often beyond the scope of standard toolkits; furthermore, due to the described peculiarities, existing solutions usually require higher levels of user involvement or suffer from limited generalizability. Besides, these techniques are highly susceptible to cell movement and deformation^8^. Furthermore, proposed combinations of traditional image processing methods^14–18^ are usually applied on a frame-by-frame basis. This bears a high risk of temporally inconsistent segmentation results for different frames in the presence of temporal intensity changes. In addition, limited generalization capability and prohibitive computational complexity pose problems for the segmentation of typically larger volumes of data recorded from different sets of cells and under different acquisition conditions.

The urge to develop generic segmentation and cell tracking algorithms, therefore, prompted the use of machine learning principles and is at the moment shaped by deep learning methods^19, 20^. Currently dominating neural networks, however, usually comprise a feed-forward architecture, trained on static and independent frames. Due to the associated risk of temporally inconsistent segmentation results, we suggest utilizing recurrent neural networks (RNNs) to take advantage of temporal correlations in the data.

The applicability of recurrent neural networks, and especially deep learning-based RNNs, is nevertheless, at the moment, limited by the difficult and computationally expensive training process, making use of techniques such as temporal back-propagation^21^. Reservoir computing (RC) provides a computationally efficient alternative framework for RNN training^22, 23^. Its properties make it interesting for the biomedical domain^24, 25^, but, so far, applications are predominantly described for other fields^22, 26, 27^, mainly focusing on signal processing tasks. In turn, RC application to (biomedical) image processing can only rarely be found^28, 29^; and we are not aware of previous work on RC-based processing of spatio-temporal image data.

### Contributions

Building on our previous work^30^, we present, to the best of our knowledge, the first study that explores the capabilities of reservoir computing in the context of segmentation of spatio-temporal image series. Specifically, we developed RC algorithms that are suitable for application to single- and dual-wavelength $\hbox {Ca}^{2+}$ imaging data and T-cell segmentation. The RC-based algorithms are compared to state-of-the-art deep learning architectures: a standard U-Net^31^ as de-facto standard in image segmentation and an U-Net-based convolutional long short term memory (LSTM)^32^ as problem-tailored state-of-the-art deep learning RNN solution.

## Materials and methods

### Reservoir computing for spatio-temporal image segmentation

**Figure 1:**
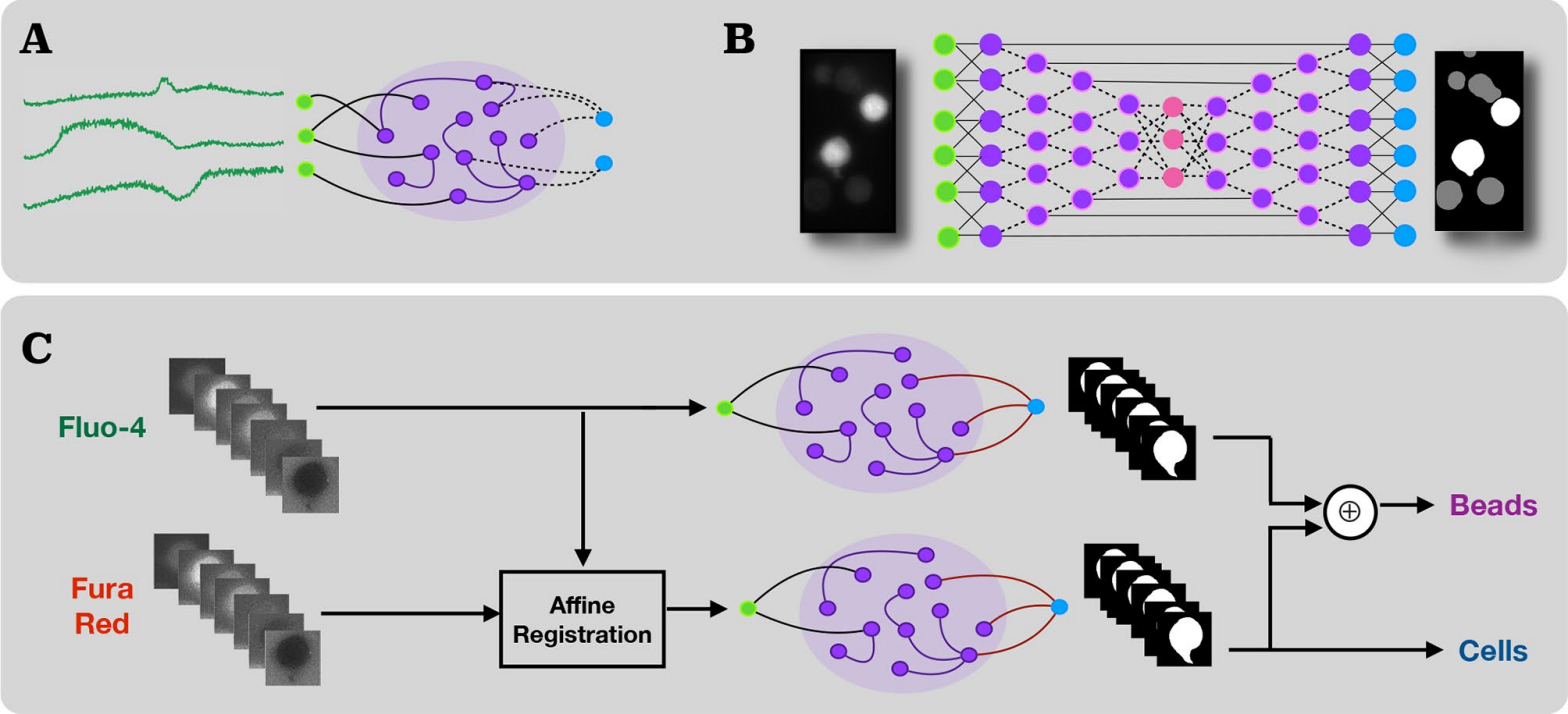
Illustration of the proposed models to segment and classify T-cells/beads in dual-wavelength $\hbox {Ca}^{2+}$ imaging data. (**A**) Schematic representation of a reservoir computing model with fixed recurrent weights (solid lines) and trainable readout weights (dashed lines) commonly used for time-series processing. In the present work, temporal image series are converted into and processed as multiple parallel time-series (shown in green) via the encoding schemes detailed in the main text. (**B**) Schematic of a deep convolutional, U-Net-like neural network (dashed lines: trainable weights). (**C**) The proposed system for T-cell/bead segmentation and differentiation (Task 2) [Graphics created with MATLAB and the Image Processing Toolbox Release 2019b, The MathWorks, Inc. (http://mathworks.com/products/image.html)].

The core to a reservoir computing model is a random, sparse, but fixed recurrent neural network, known as the *reservoir* (Fig. 1A), that non-linearly maps a time-dependent input signal into a higher dimensional signal space through the internal states of this dynamical system. The time-dependent output is computed as a linear combination of these reservoir variables. In contrast to traditional *deep* RNN training methods, RC only adapts the output weights to minimize an error measure (usually the mean squared error) between the desired target and the output signals. Thus, the neuron connections remain fixed, except for those from the reservoir toward the output layer, the so-called *readout connections*^21^. The non-linear expansion of the input signal into the high(er)-dimensional reservoir space, plus ease of training, enable RC models to efficiently learn to extract spatio-temporal features from time-dependent signals.

Using a general notation, the RC dynamics are governed by

1 $$\begin{aligned} {\mathbf {x}}(t+\Delta t) = f ({\mathbf {W}}{\mathbf {x}}(t)+{\mathbf {W}}^{in}{\mathbf {u}}(t+\Delta t)), \end{aligned}$$

with ${\mathbf {x}}(t) \in {{\mathbb {R}}}^{N_{x}}$ denoting the time-dependent $N_{x}$-dimensional reservoir state (i.e., a reservoir with $N_{x}$ units), $\Delta t\in {{\mathbb {R}}}^+$ the time sampling period, ${\mathbf {W}}\in {{\mathbb {R}}}^{N_x\times N_x}$ and ${\mathbf {W}}^{in}\in {{\mathbb {R}}}^{N_x\times N_u}$ as internal and input weight matrices, respectively, and ${\mathbf {u}}(t) \in {{\mathbb {R}}}^{N_{u}}$ the input at time *t*. The internal states are updated via the non-linear function *f*.

The output ${\mathbf {y}}(t)\in {\mathbf {R}}^{N_{y}}$ is obtained from the extended system state ${\mathbf {z}}(t) = [{\mathbf {x}}(t); {\mathbf {u}}(t)]$ with $[\cdot ;\cdot ]$ as vertical vector concatenation by

2 $$\begin{aligned} {\mathbf {y}}(t) = g({\mathbf {W}}^{out}{\mathbf {z}}(t)), \end{aligned}$$

with *g* as an output activation function and ${\mathbf {W}}^{out}\in {{\mathbb {R}}}^{N_y\times (N_x+N_u)}$ the readout weight matrix. Training the RC system then means training the readout weights (depicted by dashed lines in Fig. 1A) by computing the linear regression weights of the target outputs on the already harvested states of the reservoir units and the inputs via ridge-regression^10^.

### Encoding temporal image series into reservoir computing input data

A reservoir computing model in its standard formulation (i.e., Eq. 1) expects a single or multiple parallel time-series in the input. For temporal image processing applications, therefore, the temporal image series $\left( {\mathbf {I}}_i\right) _{i=1,\dots ,N}$, ${\mathbf {I}}_i\in {{\mathbb {R}}}^{n_1 \times n_2}$ with $n_1$ and $n_2$ as number of pixels along the image axes must be converted into corresponding RC input data ${\mathbf {u}}$. In this study, we defined the following *encoding* schemes.

#### Encoding scheme 1

Encoding scheme 1 is based on a straightforward vectorization of the images. For each image ${\mathbf {I}}_i$, six vectors ${\mathbf {i}}^{(k)}\in {{\mathbb {R}}}^{n_1n_2}$ ($k=1,\dots ,6$) were generated by ${\mathbf {i}}^{(1)}=\text {vec}\left( {\mathbf {I}}_i\right)$, ${\mathbf {i}}^{(2)}=\text {vec}\left( {\mathbf {I}}_i^T\right)$, ${\mathbf {i}}^{(3)}$ and ${\mathbf {i}}^{(4)}$ as forward- and ${\mathbf {i}}^{(5)}$ and ${\mathbf {i}}^{(6)}$ as backward-shifted versions of ${\mathbf {i}}^{(1)}$ and ${\mathbf {i}}^{(2)}$, i.e. ${\mathbf {i}}^{(3)} =\left[ 0,i^{(1)}_1,\dots ,i^{(1)}_{n_1n_2-1}\right] ^T$ and ${\mathbf {i}}^{(5)} =\left[ i^{(1)}_2,\dots ,i^{(1)}_{n_1n_2},0\right] ^T$ and similar for ${\mathbf {i}}^{(4)}$ and ${\mathbf {i}}^{(6)}$. Forward- and backward-shifting as well as vectorization of the transposed image matrix aimed at providing spatial context to the reservoir. For a temporal image sequence $\left( {\mathbf {I}}_i\right) _{i=1,\dots ,N}$, the input to the reservoir is eventually a real-valued matrix of size $7\times \left( Nn_1n_2\right)$ with the first six rows corresponding to the ${\mathbf {i}}^{(k)}$ vectors for all *N* time points, and the seventh row containing a fixed bias. Thus, for the defined encoding scheme, the variable *t* defined in Eq. (1) does *not* directly refer to the temporal index of the image series frames, but to the sequential pixel order in the vectors. The reservoir state update still follows Eq. (1) with ${\mathbf {u}}(t)$ and ${\mathbf {u}}(t+\Delta t)$ denoting the *t*^*th*^ and $(t+1)^{{th}}$ entities of the 7-dimensional input to the RC model (i.e., $\Delta t=1$).

Using this encoding scheme, the segmentation task is formalized as a supervised binary classification problem with a single output node. The output node computes a linear combination of the reservoir states and returns a real-valued vector of length $Nn_1n_2$. A threshold function is then applied to map this vector to a $\{0,1\}$-vector of the same length; the threshold represents an additional hyperparameter. The resulting binary vector is finally re-ordered into the desired binary image series of length *N*.

#### Encoding scheme 2

**Figure 2:**
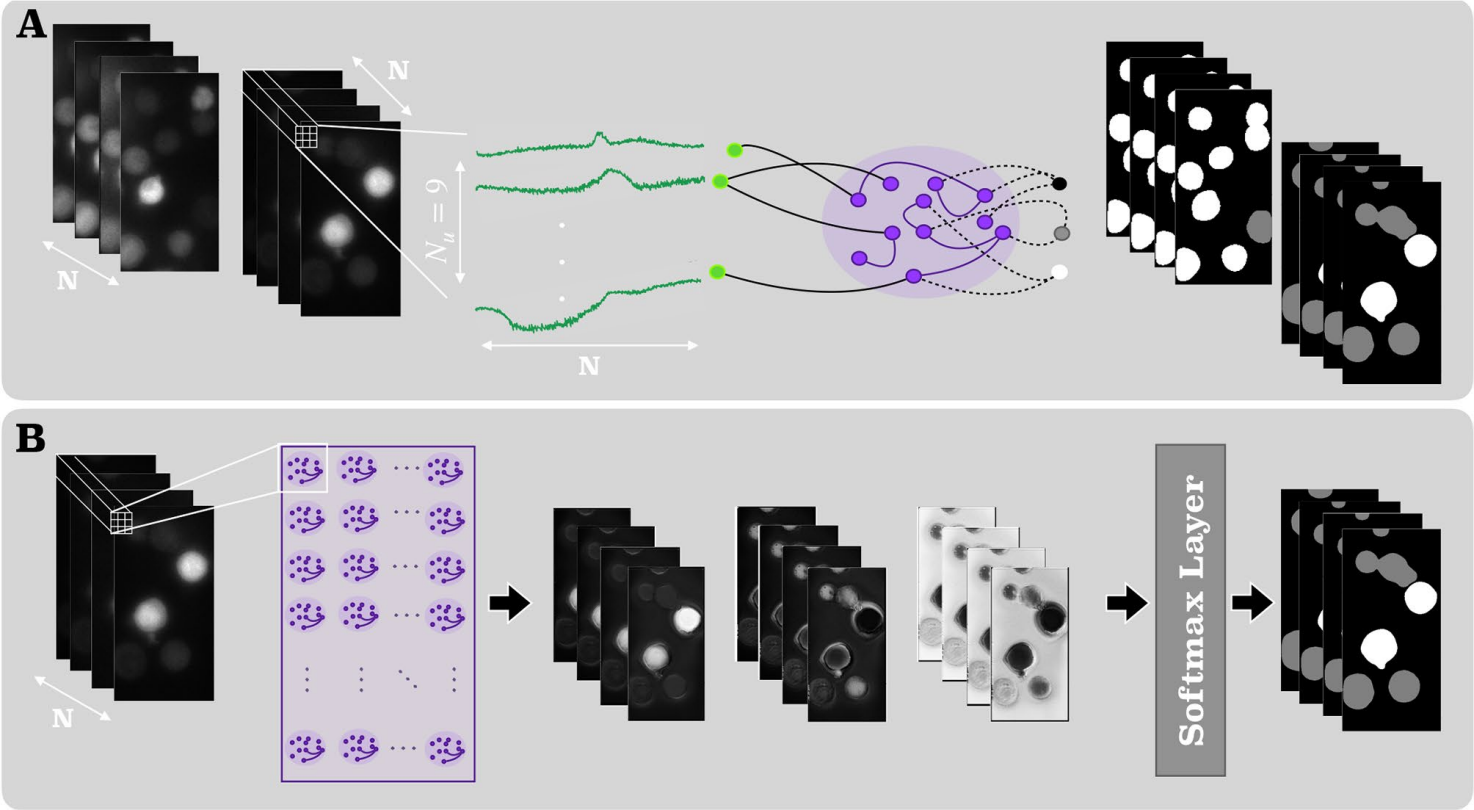
Illustration of the distributed RC structure for segmentation/classification (task 3) using encoding scheme 2. (**A**) Schematic of a single reservoir computing model trained on multitudes of $N_u=9$ univariate time-series recorded at the sites of randomly sampled adjacent pixels. The three outputs are associated with ternary output classes. (**B**) Illustration of the system functioning during model inference [Graphics created with MATLAB and the Image Processing Toolbox Release 2019b, The MathWorks, Inc. (http://mathworks.com/products/image.html)].

The encoding scheme 2 is illustrated in Fig. 2. In comparison to the straightforward encoding scheme 1, it focuses on a pixel-level analysis and aims at a denser integration of spatial information. In detail, the number of input neurons of the reservoir is chosen to be $N_u=9$, covering the intensity information of a $3\times 3$ pixel neighborhood of a pixel for each time point of an image series of length *N*. Different to encoding scheme 1, in this case, variable *t* of Eq. (1) indeed refers to the temporal index, i.e. the frame number, of the considered image time series; ${\mathbf {u}}(t)$ and ${\mathbf {u}}(t+\Delta t)$ denote to the pixel intensities in the $3\times 3$ neighborhood of the processed pixel at *t* and $t+1$. Applied in a three-class segmentation context (see “Task 3: T-cell/bead segmentation and classification in single-emission measurements”), the encoding scheme is applied together with a RC model with a three-neuron output layer, returning the class-specific RC outputs for the considered pixel at a specific time point of the time series. RC inference for all image pixels leads to three temporal image series with *N* frames, which are converted into probability values via softmax layers. Based on the class-specific softmax values, eventually, *N* ternary images are generated.

### Image acquisition and data characteristics

The image data in this study were acquired by live-cell fluorescence microscopy as detailed by Diercks et al.^33^.

Briefly, imaging was carried out with a Leica IRBE2 microscope (100-fold magnification) using a Sutter DG-4 as a light source at the image acquisition frequency of 40 Hz (data acquisition with Hamamatsu C9100 EMCCD camera). A dual-view module (Optical Insights, PerkinElmer Inc.) was used to split the emission wavelengths of the two imaged $\hbox {Ca}^{2+}$ indicators^4^.

Our experiments focused on Jurkat T-cells that typically exhibit significant motion and deformation during imaging, allowing us to better illustrate the advantages of the proposed segmentation algorithms. In addition, primary T-cell data were used to analyze generalizability capabilities of RC-based cell segmentation models. Primary T-cells are typically smaller and, from that perspective, more challenging to segment than Jurkat T-cells. They, however, exhibit less motion and deformation than Jurkat T-cells and are, therefore, less suited for analysis and illustration of the impact of integration of temporal information into the segmentation process.

**Figure 3:**
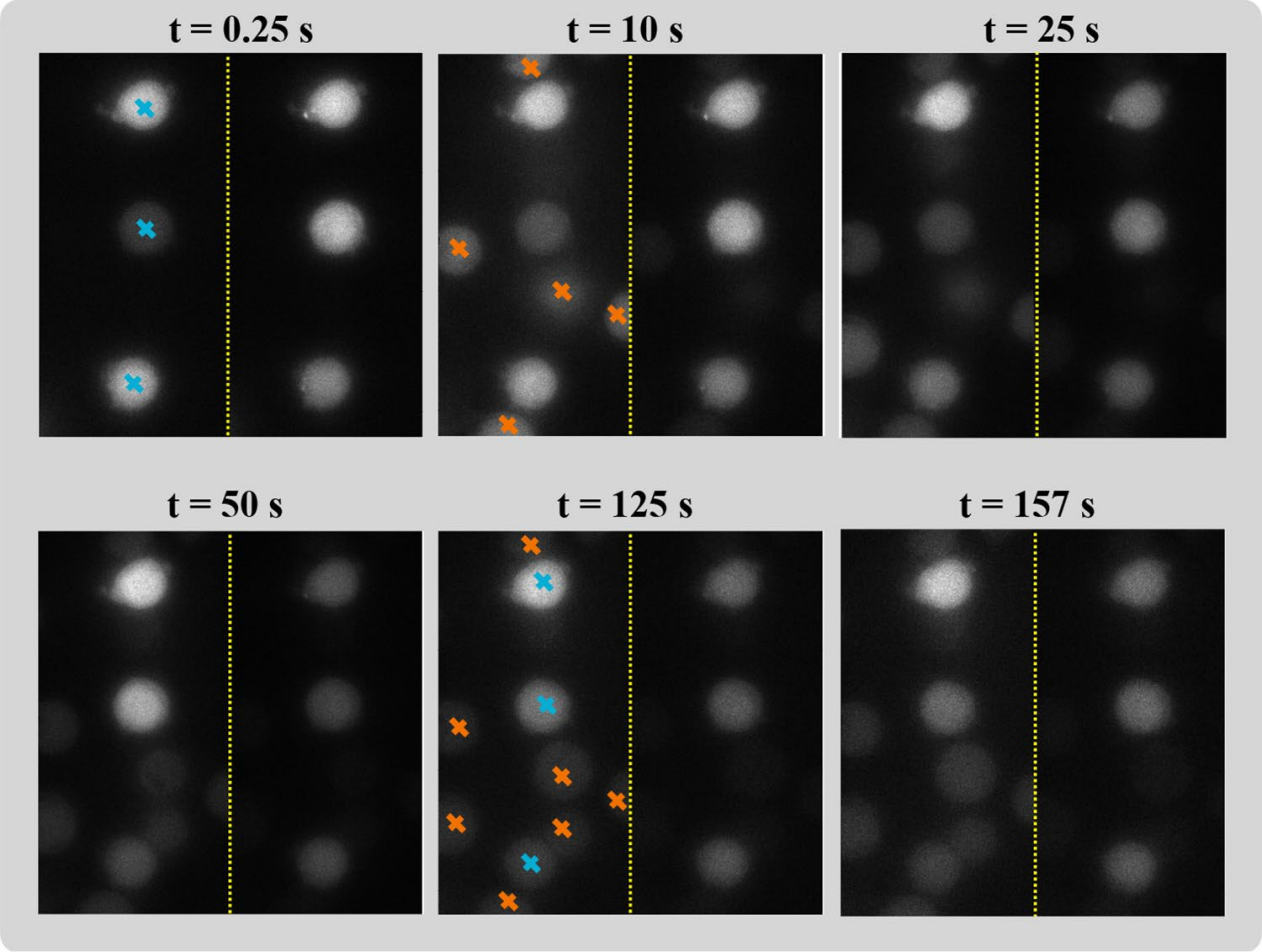
Six snapshots (with $500 \times 500$ pixels) of fluorescence intensity measured with Fluo-4 (left to the yellow border) and Fura Red (right) emissions. Cells are marked with blue crosses and beads with orange crosses at first appearance and, for further clarification, at t = 125 s. While there is no bead at t = 2.5 s, at t = 10 s, five and at t = 25 s, six beads are observable in the Fluo-4 images. The beads are not captured by Fura Red. Visible in all Fura Red images, three Jurkat T-cells with different sizes, shapes, and temporal activities are present [Graphics created with MATLAB and the Image Processing Toolbox Release 2019b, The MathWorks, Inc. (http://mathworks.com/products/image.html)].

All cells were loaded with Fluo-4 and Fura Red as cytosolic $\hbox {Ca}^{2+}$ indicators and stimulated by beads coated with CD3-antibodies. The beads were added after several seconds of image acquisition. A typical image frame had a size of $500 \times 250$ pixel with a spatial resolution of 368 nm for each emission-wavelength; the resulting temporal sequence comprised $>7000$ frames. Example data are shown in Fig. 3.

### Application scenarios and experiments

We focused on three segmentation scenarios: (1) single object segmentation; (2) T-cell and bead segmentation and differentiation exploiting two emission-wavelengths information; and (3) T-cell/bead segmentation and differentiation in single emission-wavelength recordings. In the following, the developed RC algorithms are detailed and corresponding experiments described. The experiments were based on ten live-cell imaging recordings (computer hardware: Intel Xeon(R) E-2186 (3.80 GHz), 32 GB RAM, NVIDIA GeForce RTX 2080 Ti).

#### Task 1: single object segmentation

The first task aimed at illustrating general feasibility and an initial evaluation of RC-based object segmentation in spatio-temporal microscopy data. Given manually extracted regions of interest (ROIs) that include a single object and a set of sequential frames of the object, generic RC models were implemented to segment the object. Segmentation was performed on single emission-wavelength data, i.e., either Fluo-4 or Fura Red imaging data.

Reference segmentation (ground truth, GT) data for evaluation purposes was generated semi-manually. An unsupervised RC-based clustering model was trained for cell-customized pixel-wise data annotation. The model suggestions were visually presented to a human observer and rated as “well-labeled” or “bad”, reducing the laborious manual pixel-wise labeling to a binary classification task. The GT generation process (presented in Fig. S1) is detailed in Supplementary Note 1. 2000 ROIs with Jurkat T-cells (40 cells with 50 frames each; ROI size: $128\times 128$ pixel) that were judged “well-labeled” were used for subsequent model training and evaluation (Fig. S2 illustrates samples of frames marked as successfully labeled). A subset of 280 frames (only Fluo-4 emission) was also manually re-labeled. The manually annotated data were used to investigate whether the GT generation process resulted in a potential bias toward overestimation of RC segmentation accuracy. Finally, a set of 22 primary T-cells (50 frames each; same ROI size than for the Jurkat T-cells) were used to test whether the trained RC model is able to deal with imaging data for a different cell type not seen during training.

The RC hyperparameters were optimized by 5-fold cross validation using 500 of the 2000 ROIs (i.e., 10 cells; final parameters: $N_x=100$, each neuron randomly connected to 10 neurons; activation function: tanh) based on encoding scheme 1. After parameter selection, the final RC model was trained on the entire 500 ROIs.

RC segmentation performance was compared to the U-Net (Fig. 1B; here: with a ResNet34-pretrained encoder^34^) and a state-of-the-art deep learning RNN architecture for cell segmentation that integrates the idea of convolutional long short-term memory networks (C-LSTM) into the U-Net by substituting the standard convolutional layers of the encoder with C-LSTM layers that offer recurrent connections^32^. Training of the deep learning systems (hyperparameters were the default parameters) was performed on the same 500 ROIs used for RC training. As an additional classical baseline, we also applied Otsu thresholding.

The segmentation approaches were evaluated in detail on the 1500 ROIs and 30 Jurkat T-cells that were not used during training. The generalization capability of the trained RC model was further investigated on the above-mentioned 22 primary T-cells and the corresponding 1100 frames. Segmentation accuracy measures were pixel-wise accuracy and the Dice coefficient^35^.

To also investigate the hypothesis that consideration of temporal correlations in the data as done in RNN-based models helps improving temporal consistency of segmentation results, a contour evolution analysis was performed. Therefore, the perimeter of the segmentation masks, its orientation (angle between the image x-axis and the major axis of the cell), and the mask area were evaluated for different models and the Jurkat T-cell data.

#### Task 2: T-cell/bead segmentation and differentiation using two-emission-wavelengths measurements

To demonstrate transferability of the task 1 results to a ‘real-world’ scenario, task 2 addresses the segmentation of full frames and temporal image data that contain multiple T-cells and antibody-coated beads. Thus, segmentation of T-cells not only means to reliably segment high intensity objects, but also to differentiate between T-cells and beads. Viewed in a single frame and emission wavelength (i.e., Fluo-4 or Fura Red), cells and beads can hardly be differentiated even by human observers (Fig. 3). The profound gradient between bead intensity values of corresponding Fluo-4 and Fura Red imaging data, however, can ameliorate the segmentation performance when the system is provided with the information of both cytosolic $\hbox {Ca}^{2+}$ indicator emissions.

Therefore, suitable to be integrated into dual-wavelength $\hbox {Ca}^{2+}$ imaging systems, we propose the RC-based segmentation and object classification scheme outlined in Fig. 1C. The system comprises two trained reservoir models: one reservoir directly receives $\hbox {Ca}^{2+}$ images from Fluo-4 measurements, and the other one is presented with Fura Red sequences, affinely registered to the corresponding Fluo-4 frame to compensate for a potential misalignment of the different emission wavelength imaging information. Methodically, the two RC systems are identical to the approach described in “Task 1: single object segmentation”. Subsequent to object segmentation in both emissions, a logical XOR operation is applied to discriminate cells and beads.

Similar to task 1, a semi-manual RC-based annotation system (depicted in Fig. S3) was implemented to create ternary images (classes: background, T-cell, bead) and GT data as described in Supplementary Note 2. Example GT data are shown in Fig. S4. RC training was based on 1155 full-size frames (231 frames from 5 Jurkat T-cell image series, each frame with a size of $500\times 250$ pixel; RC hyperparameters like in task 1, but $N_x=500$). Testing was performed on a separate set of 1155 frames from five different Jurkat T-cell image series (frame number chosen due to RAM limitations).

The performance of the proposed RC algorithm was again compared to U-Net and U-Net-based LSTM results. To ensure comparability, the deep learning approaches were set up similar to the RC system: two models were trained, one using Fluo-4 and one using Fura Red information and the results combined via XOR. Training and test data were the same as used for the RC.

#### Task 3: T-cell/bead segmentation and classification in single-emission measurements

Simultaneous imaging of two $\hbox {Ca}^{2+}$ indicators like Fluo-4 and Fura Red is motivated by the advantages of dual-wavelength ratiometric fluorescence microscopy: Computing the ratio between corresponding fluorescence intensity values allows, e.g., correcting for artifacts due to locally varying dye concentration, variations in laser intensity, and calculation of absolute $\hbox {Ca}^{2+}$ concentrations^36^. Furthermore, being able to use one excitation wavelength for two $\hbox {Ca}^{2+}$ indicators has the advantage to detect local $\hbox {Ca}^{2+}$ microdomains at a very high temporal and spatial resolution^9^.

However, aiming, for instance, at identification of players involved in the development of initial $\hbox {Ca}^{2+}$ microdomains, a correlation of increased local cytosolic $\hbox {Ca}^{2+}$ microdomains to cell organelles and $\hbox {Ca}^{2+}$ channels is desirable, requiring staining the structures and measuring the corresponding fluorescence signal. For such scenarios, it is common to image the intracellular $\hbox {Ca}^{2+}$ concentration using only a single $\hbox {Ca}^{2+}$ indicator like Fluo-4. This, in turn, means that algorithms are required to differentiate T-cells and antibody-coated beads without extra information from other recording emission-wavelengths such as Fura Red in task 2.

**Figure 4:**
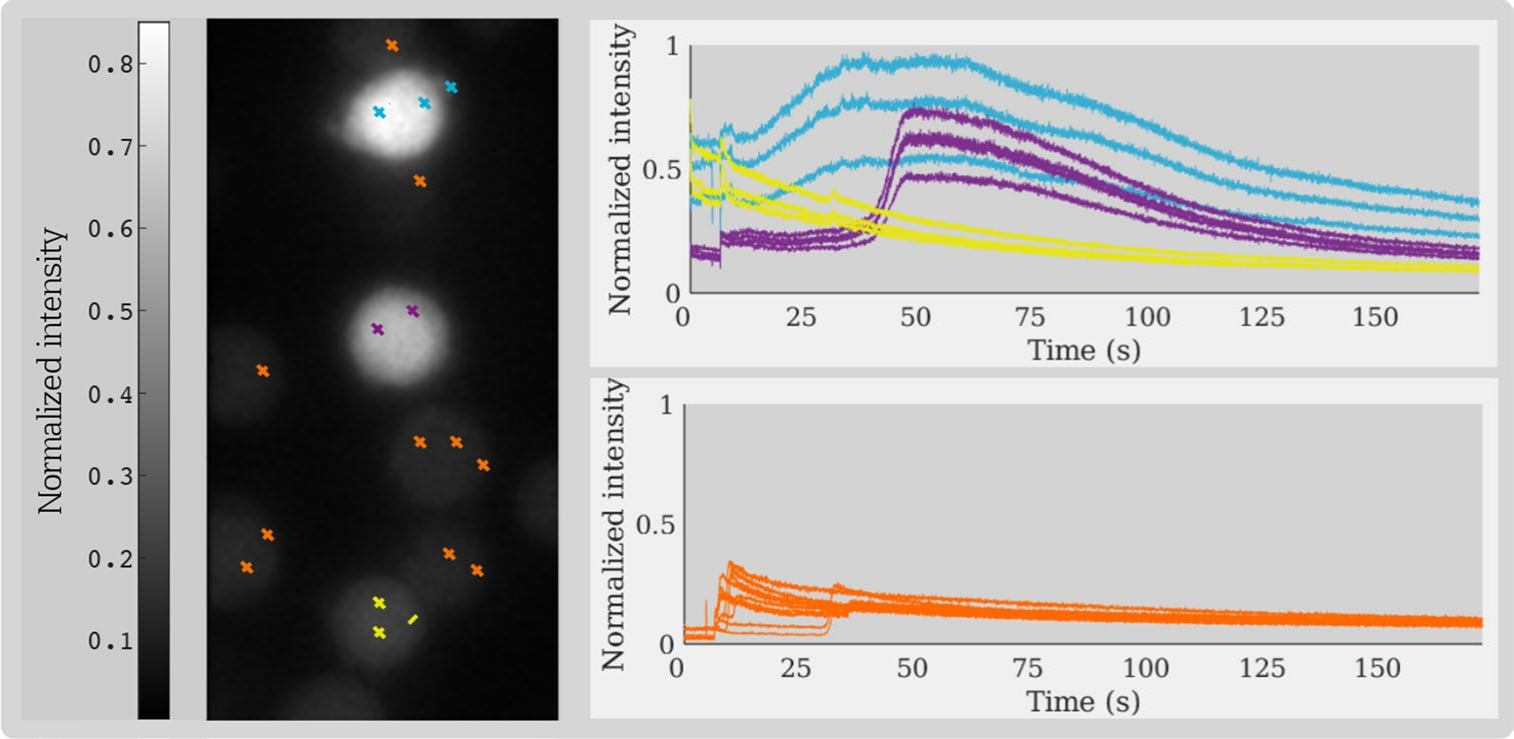
Examples of temporal signals recorded for Jurkat T-cell pixels (depicted in blue, purple and yellow) and antibody-coated beads (orange). The time-series highlight the notable difference in the fluorescence intensity levels obtained from different cells. Although the graphs recorded from sites corresponding to beads follow similar temporal patterns, there are lags between the times they appear in the imaging data. Near the end of the recording course, the fluorescence intensity at the bead sites are quite comparable to the cell intensities, making a frame-by-frame segmentation/classification ineffective [Graphics created with MATLAB and the Image Processing Toolbox Release 2019b, The MathWorks, Inc. (http://mathworks.com/products/image.html)].

To illustrate the complexity of this task, the temporal intensity profile of different cell and bead pixels are plotted in Fig. 4. The differentiation of cells and beads becomes almost impossible if taking into account the image information of only a single frame. The hypothesis is that RNNs are able to perform the task by making use of the distinct temporal patterns for bead and cell pixels.

To tackle the task by RC, we first re-used and evaluated the RC system and encoding scheme 1 as described for task 2. Further, to enforce the RC system to better preserve local spatial correlations of the data while simultaneously focusing on temporal pixel intensity patterns, we implemented and applied encoding scheme 2 (see “Encoding temporal image series into reservoir computing input data”).

For the first RC system and encoding scheme, the training data was similar to the one used in task 2, except for using only the Fluo-4 imaging data. For the second RC encoding scheme, $20\times 10^7$ pixels from the same data were selected for training. Hyperparameters were kept similar to the first encoding scheme, except for replacing the tanh activation function by ReLU. The test dataset was the same used for task 2.

The performance of the RC algorithms was compared to the results obtained by the standard U-Net and an adaptation of the U-Net-LSTM for multi-class classification.

Table 1 summarizes the RC input data characteristics and reservoir parameters for the individual tasks. For all tasks, the outputs of the different segmentation algorithms were post-processed following Arbelle et al.^32^ (i.e., application of morphological hole closing, removal of small segmented clusters) to avoid holes within the segmented objects and to reduce the number of false positive pixels. The post-processing parameters were identical for all segmentation approaches.

**Table 1:** Summary of the characteristics of the input data used in the present study and the chosen reservoir parameters for individual tasks.

	Input data characteristics		Reservoir parameters		
	Total number of frames per image series	Frame size	Number of neurons	Activation function *f*	Readout function *g*
Task 1	50 (each containing a single cell)	$128 \times 128$	100	tanh	Identity
Task 2	231 (entire image frames)	$500 \times 250$	100	tanh	Identity
Task 3	231 (entire image frames)	$500 \times 250$	500	ReLU	Sigmoid

## Results

### Single object segmentation

**Figure 5:**
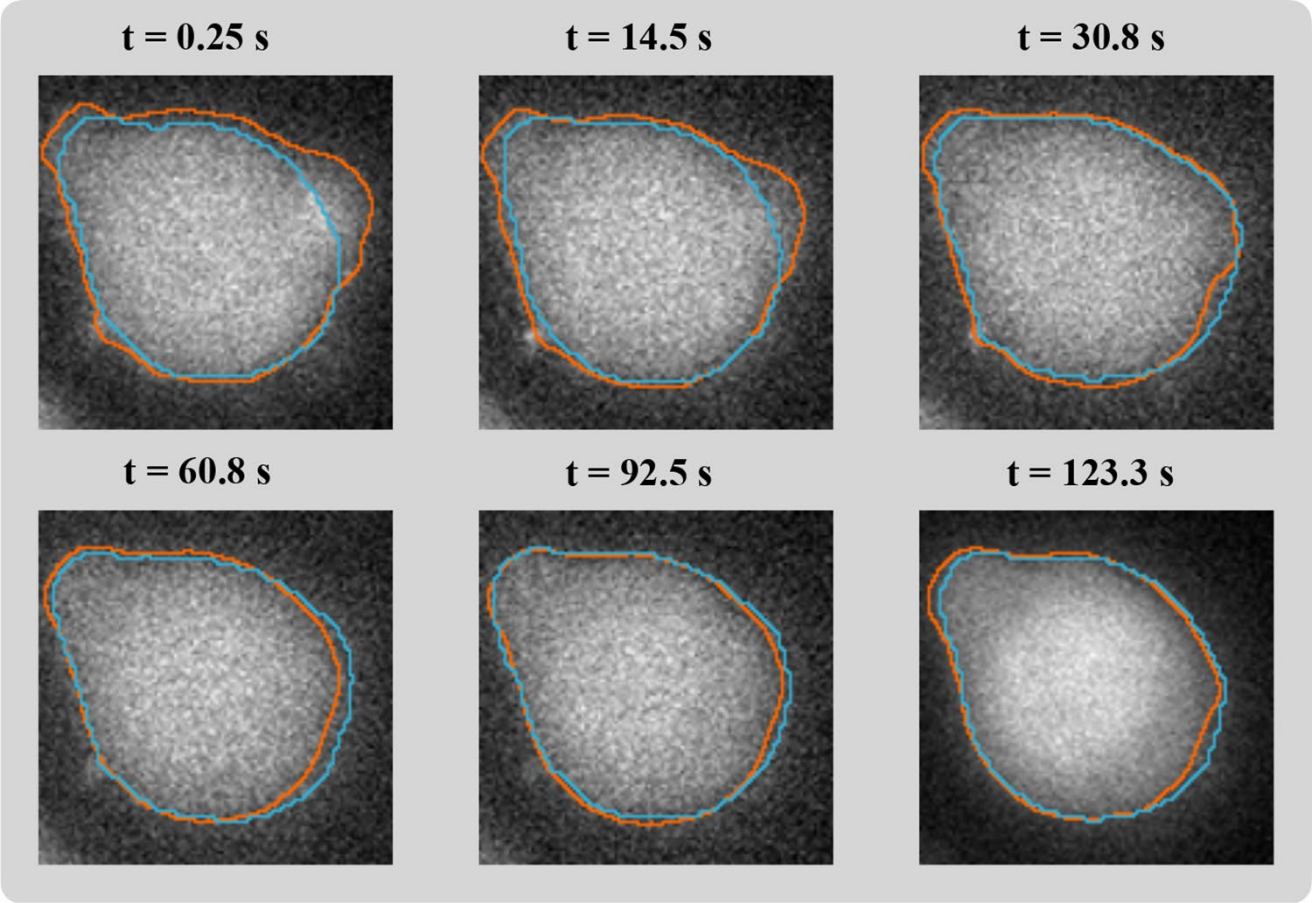
Illustration of task 1 results: Jurkat T-cell segmentation for six frames of a Fluo-4 $\hbox {Ca}^{2+}$ imaging sequence. Blue: segmented mask border for the standard U-Net model. Orange: cell borders obtained by the reservoir computing model [Graphics created in MATLAB and the Image Processing Toolbox Release 2019b, The MathWorks, Inc. (http://mathworks.com/products/image.html)].

Figure 5 shows segmentation results for an exemplary Jurkat T-cell and six frames for the RC algorithm and a standard U-Net. Both approaches achieve a fairly good segmentation quality. For some frames, the U-Net segmentation is closer to the visually perceived cell border, and for some, the RC results appear more appropriate. The same holds true for a comparison to the U-Net-LSTM. This visual impression is also reflected by the quantitative evaluation summarized in Table 2 (upper part): For the full test dataset, all machine learning-based segmentation approaches achieved accuracy values between 0.94 and 0.95 and Dice coefficients between 0.92 and 0.93 for both cytosolic $\hbox {Ca}^{2+}$ indicator emissions (differences between algorithms or emissions not significant; testing by two-sample t tests; $p > 0.31$ for all comparisons). In comparison, the Dice values for Otsu thresholding, applied as a classical baseline approach, were between 0.89 and 0.91 ($p>0.08$ for comparisons to the other segmentation approaches).

The sub-analysis on the potential bias due to the GT generation process is summarized in Table 2 (lower part). There exist no significant difference between the metrics values for the semi- and the entirely manually annotated data that would indicate existence of a bias.

**Table 2:** Quantitative evaluation of the segmentation accuracy of the considered machine learning models for **task 1**: reservoir computing, a standard U-Net, and an U-Net-based LSTM (evaluation metrics: mean class-wise accuracy and Dice coefficient).

		Accuracy	Dice value
Evaluation using full semi-manual test set			
RC	Fluo-4	0.9453	0.9321
	Fura Red	0.9446	0.9315
U-Net	Fluo-4	0.9447	0.9297
	Fura Red	0.9415	0.9265
U-Net-LSTM	Fluo-4	0.9459	0.9321
	Fura Red	0.9369	0.9232
Otsu thresholding	Fluo-4	0.9402	0.8902
	Fura Red	0.9371	0.9096
Comparison of performance for manual/semi-manual subset			
RC	Manual	0.9242	0.9102
	Semi-manual	0.9252	0.9131
U-Net	Manual	0.9397	0.9233
	Semi-manual	0.9383	0.9232
U-Net-LSTM	Manual	0.8929	0.8748
	Semi-manual	0.8995	0.8862

As a classical baseline approach, segmentation results for standard Otsu thresholding are also listed for the semi-manually generated test set. This test set consisted of 1500 frames captured from 30 Jurkat T-cells, i.e., 50 frames/cell. The subset used for the investigation of a potential bias due to the proposed semi-manual GT generation approach comprised a subset of 280 frames that were also manually labeled ($\hbox {Ca}^{2+}$ indicator: Fluo-4).

**Figure 6:**
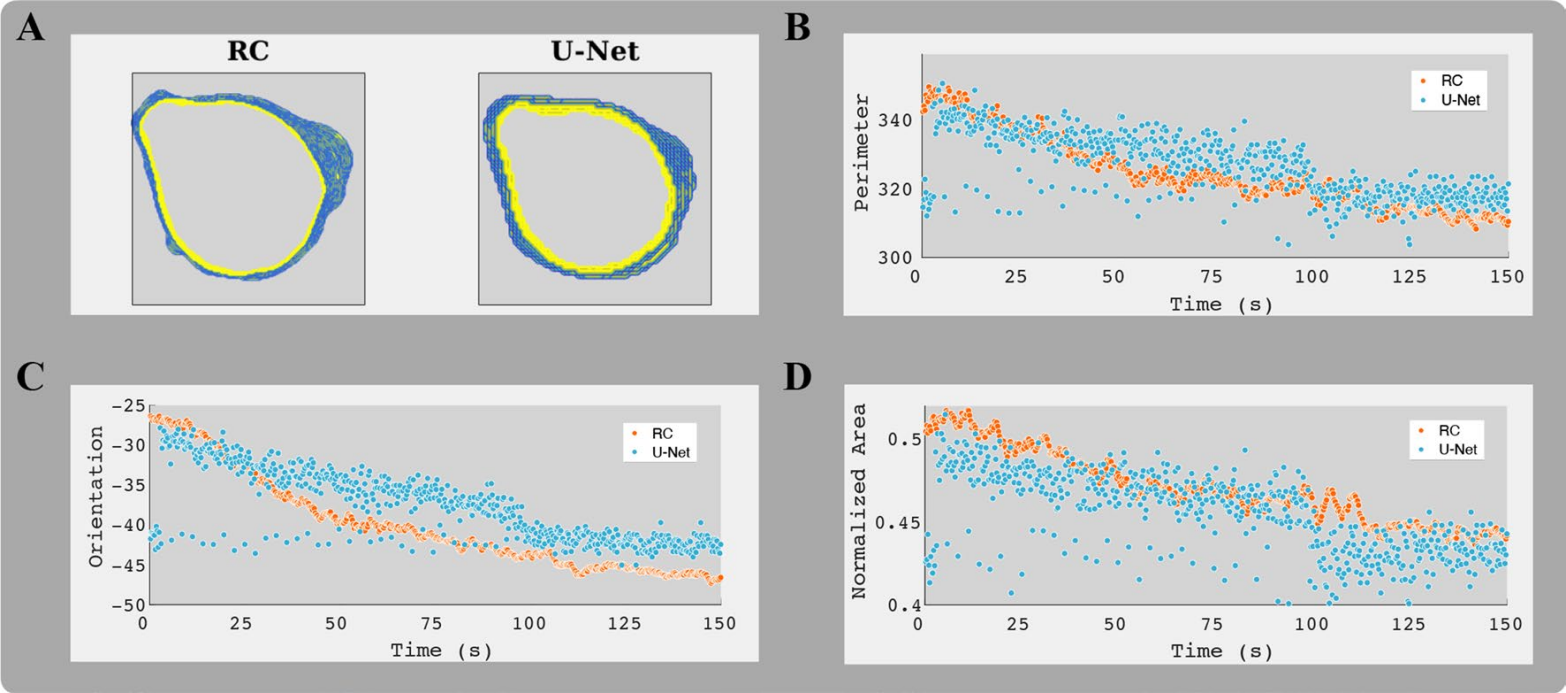
Predicted temporal contours evolution for a representative Jurkat T-cell and segmentation in the Fluo-4 emission data. (**A**) Unlike the discrete contour trajectory returned by the standard U-Net, the contours predicted by the RC method evolve continuously. (**B**) Frame-to-frame changes in contour length (in number of pixels) of the predicted masks. (**C**) Mask orientation, the angle (in degree) between the horizontal image axis (i.e., x-axis) and the major axis of an ellipse with the same second-moments as the segmented object, over time. (**D**) Normalized mask area over time. The area of the object was divided by the total number of pixels in each frame. The same illustration for the results of the U-Net-LSTM is presented in the supplementary materials (Fig. S5) [Graphics created with MATLAB and the Image Processing Toolbox Release 2019b, The MathWorks, Inc. (http://mathworks.com/products/image.html)].

The results of the analysis of the temporal consistency of the segmentation masks are illustrated in Fig. 6 and supplemental video S1. The incorporation of temporal information by the RC system leads to smoother contour trajectories. While for the U-Net (i.e., frame-by-frame segmentation), the contour length, the mask orientation, and the mask area show abrupt changes between different frames, respective measures for the RC system show a smoother and more plausible evolution. For the deep learning-based RNN approach (U-Net-LSTM), the results are similar to the RC system (presented in Fig. S5, supplementary document).

Application of the RC model trained for segmentation of Jurkat T-cells to unseen primary T-cells led to a drop of the Dice values compared to Jurkat T-cell segmentation. For Fluo-4 emission measurements, the accuracy and the Dice value were 0.9476 and 0.8137, respectively; for Fura Red emission data, the accuracy and the Dice value were 0.9674 and 0.8670. The Dice values indicate a potential overfitting of the RC model to Jurkat T-cell data characteristics. However, Otsu thresholding applied to the same data yielded Dice values of 0.7189 (Fluo-4; $p=0.004$ for comparison to RC Dice values) and 0.8138 (Fura Red; $p=0.008$). Thus, the information learned by the RC model appears helpful compared to the basic intensity-based two-class pixel differentiation even for the different cell type data.

### T-cell/Bead segmentation and differentiation using two-emission-wavelenghts measurements

The results for task 2 are summarized in Table 3. Given the different appearance of the beads for the two emission wavelengths, this task appears relatively straight-forward. However, the accuracy and Dice values indicate that incorporation of temporal information already helps improving segmentation and classification performance for this task: While the RC and the U-Net-LSTM systems perform on par, they both outperform the standard U-Net that was applied frame-by-frame (statistical testing omitted due to limited number of independent samples, i.e., five imaging sequences). In addition, the segmentation of the beads appears to be more complex for all three systems, with the standard U-Net almost entirely failing.

**Table 3:** Performance evaluation of the reservoir computing model and the deep learning models for **task 2** (test set based on 1155 frames extracted from five $\hbox {Ca}^{2+}$ imaging sequences).

	Objects	Accuracy	Dice value
RC	T-cells	0.9653	0.8795
	Beads	0.9518	0.5819
U-Net	T-cells	0.9367	0.8164
	Beads	0.9437	0.1389
U-Net-LSTM	T-cells	0.9631	0.8842
	Beads	0.9483	0.5836

### T-cell/bead segmentation and classification using single-emission measurements

The quantitative results for task 3 are summarized in Table 4. The standard U-Net was, similar to task 2, not able to provide acceptable results and was discarded from further analyses.

**Figure 7:**
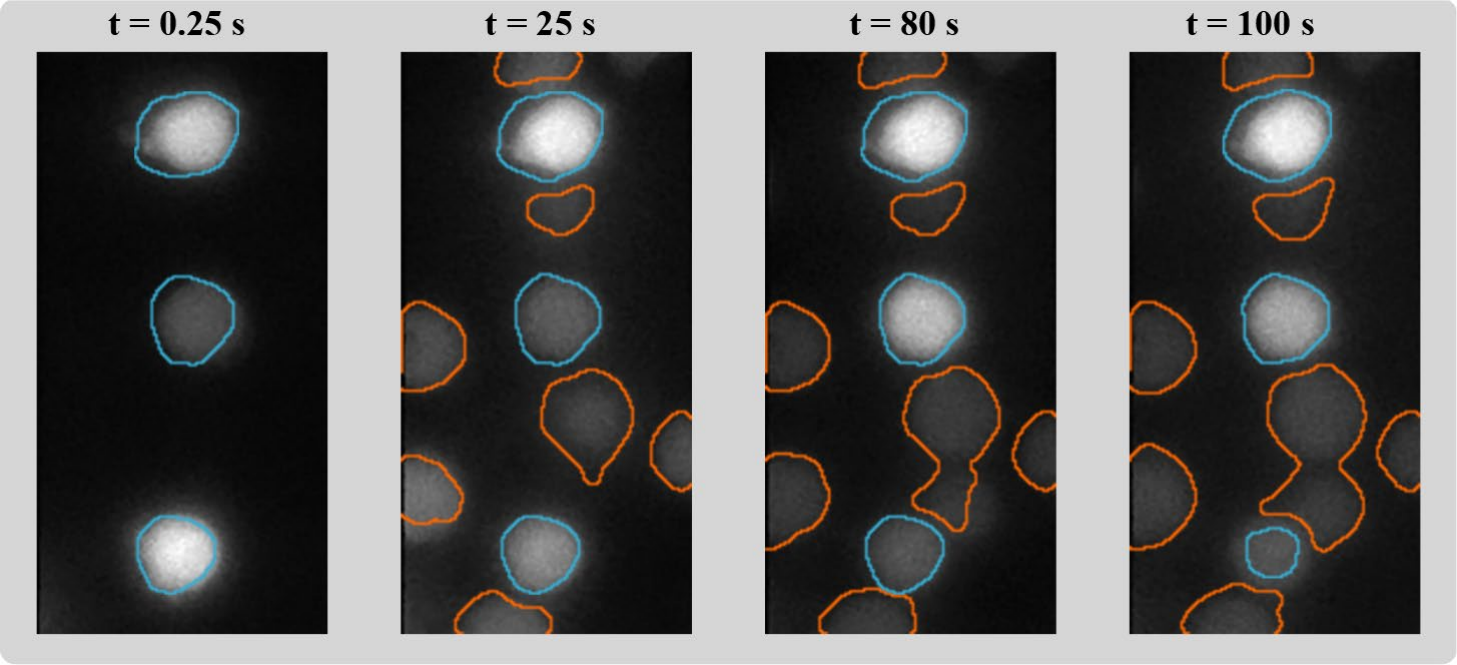
Boundaries of segmented Jurkat T-cells (blue) and anti-body coated beads (orange) in four frames of Fluo-4 $\hbox {Ca}^{2+}$ imaging data, computed by the proposed RC model with encoding scheme 2. Despite cellular and beads movement and significant local and temporal variation of fluorescence intensity, the algorithm provides a robust and efficient solution to the task [Graphics created with MATLAB and the Image Processing Toolbox Release 2019b, The MathWorks, Inc. (http://mathworks.com/products/image.html)].

Table 4 illustrates that the RC encoding style, i.e., the approach to convert the images into a format that is suitable for RC-based processing, plays an important role. Compared to the straightforward encoding scheme 1 that is based on direct vectorization of the image matrices, the second scheme led to a significant increase of segmentation accuracy. Exemplary RC (with encoding scheme 2) segmentation results are shown in Fig. 7 and the supplementary video S2. A separation of close-by beads is, however, not always feasible; this remains for further methodological refinement.

Compared to the RC models, the mean accuracy and Dice values for the U-Net-LSTM are, although being in the same range, slightly higher. It should, however, be kept in mind that the implemented RC models have a drastically lower number of trainable parameters (approximately 1500 in the current study) than the U-Net-LSTM ($> 6.5 \times 10^{6}$) and the standard U-Net ($> 3.6 \times 10^{9}$). In the current experiment, this led to a reduction of training time from 26 h for the U-Net-LSTM to 1 h for the RC system, although the U-Net-LSTM training was performed on GPU and was already highly optimized for GPU usage, while the RC training was on CPU and was not optimized for parallel computing.

**Table 4:** Performance evaluation of reservoir computing models and the U-Net-based LSTM for Jurkat T-cell and bead segmentation and differentiation in single-emission recordings (**task 3**; test set: 1155 frames from five imaging sequences).

	Objects	Accuracy	Dice value
RC (encoding 1)	T-cells	0.9226	0.7623
	Beads	0.9266	0.3534
RC (encoding 2)	T-cells	0.9520	0.8528
	Beads	0.9459	0.5909
U-Net-LSTM	T-cells	0.9497	0.8540
	Beads	0.9579	0.6242

Corresponding class-wise normalized confusion matrices are presented in Fig. S6.

## Discussion

High-resolution $\hbox {Ca}^{2+}$ imaging methods allow characterizing spatio-temporal dynamics of initial $\hbox {Ca}^{2+}$ signaling in T-cells, a fundamental process in the adaptive immune system. The increasing amount of acquired data results in a need for efficient image processing and analysis solutions. The present study explores the potential of reservoir computing (RC) for temporally consistent object and, in particular, T-cell segmentation in spatio-temporal $\hbox {Ca}^{2+}$ imaging data. The underlying rationale was that RC represents a computationally efficient RNN-based approach to learn spatio-temporal features and can help to overcome drawbacks of current deep learning systems.

Applied to Jurkat T-cell segmentation as well as bead and cell segmentation and classification using either single- or two-emission wavelengths imaging information, the RC models perform in terms of segmentation accuracy at least on par with the de-facto standard in biomedical image segmentation, the standard U-Net. For differentiation of T-cells and beads, which requires integration of temporal information, RC outperforms the U-Net, demonstrating the potential of spatio-temporal learning inherent to the RC paradigm.

Compared to a U-Net-based LSTM as a state-of-the-art RNN architecture, the RC models show a similar performance both in terms of segmentation accuracy and temporal consistency of the segmentation results. At this, it should be noted that the semi-manual GT generation pursued in the present study included a frame-by-frame visual quality check and application of frame-specific thresholds. This partly led to temporal inconsistencies of the annotations for the temporal images series used for system training (see supplemental video S2). The obtained temporally mainly consistent segmentation results therefore also illustrate a certain degree of robustness of both RNN approaches with respect to corresponding training data imperfections. However, convolutional LSTMs are computationally expensive, difficult to train, and, consequently, still rarely applied in biomedical context. In the current work, the LSTM training took, for instance, more than a day on GPU, while RC training required one hour on CPU. Furthermore, the RC model comprised 1500 trainable parameters—whereas the LSTM $> 6.5 \times 10^{6}$ parameters. Thus, a similar segmentation performance was achieved with only $0.023\%$ of trainable parameters.

Despite faster training, due to the current CPU implementation, the proposed RC-based image segmentation is, however, not real time-capable: RC inference for a single 128 $\times$ 128 pixel frame takes approximately 0.5 s for the described hardware, and the inference time scales with the number of pixels. Optimization for parallel computing and re-implementation for GPU usage is, nevertheless, expected to result in a significant shortening of RC inference times especially for the proposed encoding scheme 2, rendering real-time RC segmentation realistic.

With regard to the presented results, we would like to note that the spatio-temporal Jurkat T-cell image series considered in our study are representative for the imaging conditions and data characteristics at our laboratory^4, 9, 33^. However, it remains to be shown that our methods and observations can be transferred to and confirmed for different, maybe larger or more heterogeneous datasets and data acquired under different imaging conditions. In particular altered imaging conditions, but also cell and cell dynamics characteristics not present in the training data will necessitate retraining the models. This becomes already evident by the drop of the Dice values seen for segmentation of primary T-cells by means of the RC model trained for Jurkat T-cell segmentation (see results for task 1). To foster testing of the proposed approaches on other datasets, we provide the RC source code, together with the models and example data, as open source (see Data Availability statement).

For future work from a method perspective, it remains to extend our RC architecture by additional reservoir layers to extract multiple-scale temporal and/or spatial features. We expect the incorporation of multi-scale information to further improve segmentation accuracy.

## Conclusions

The current work demonstrates reservoir computing to be an efficient alternative to computationally expensive deep learning-based networks for temporally consistent cell segmentation in high-resolution live-cell $\hbox {Ca}^{2+}$ imaging.

## Supplementary Information

Supplementary Information 1

Supplementary Information 2

Supplementary Information 3

## Data Availability

The RC source code, the models, and example data are provided publicly available at https://github.com/IPMI-ICNS-UKE/Jurkat_cell_segmentation.
