# Supplementary material for: Spatio-temporal feature learning with reservoir computing for T-cell segmentation in live-cell Ca2+ fluorescence microscopy
Source: Sci Rep. 2021 Apr 15;11:8233. doi: 10.1038/s41598-021-87607-y (PMC8050068; doi:10.1038/s41598-021-87607-y)
Supplement: Supplementary file 1 — Supplementary Information 1 [file 41598_2021_87607_MOESM1_ESM.pdf]

Spatio-Temporal Feature Learning with Reservoir Computing for T-Cell Segmentation in Live-Cell Ca^{2+} Fluorescence Microscopy:

— Supplemental Document —

FATEMEH HADAEGHI[1,*], BJÖRN-PHILIPP DIERCKS[3], DANIEL SCHETELIG[1,2], FABRIZIO DAMICELLI[1], INSA M.A. WOLF[3], AND RENÉ WERNER[1,2]

[1] *Department of Computational Neuroscience, University Medical Center Hamburg-Eppendorf, Hamburg, Germany*
[2] *Center for Biomedical Artificial Intelligence (bAIome), University Medical Center Hamburg-Eppendorf, Hamburg, Germany*
[3] *Department of Biochemistry and Molecular Cell Biology, University Medical Center Hamburg-Eppendorf, Hamburg, Germany*
[*] *Corresponding author: Fatemeh Hadaeghi, f.hadaeghi@uke.de*

1. SUPPLEMENTARY NOTE 1: SEMI-MANUAL PIXEL-WISE DATA ANNOTATION APPROACH IN TASK 1

Our pixel-wise data labeling method (outlined in Figure S1) integrates a reservoir computing model with a dimension reduction layer to provide an accurate semi-manual ground truth generation system that relies on spatio-temporal dynamics of recorded Ca^{2+} signals. Upon receiving each imaging sequence, in order to compensate for a potential shift between the Fluo-4 and Fura Red imaging data induced by the microscope setup, an affine intensity-based registration algorithm is initially adopted to align Fura Red (as moving image) with the Fluo-4 (as the reference image) image information. At every single point in time, both the warped Fura Red and the unchanged Fluo-4 images are encoded into 1D image vectors (or "spatial sequences") by concatenating their consecutive columns. Reading through the two imaging sequences, from the first to the last frame, two large "spatio-temporal sequences" are formed from the concatenation of consecutive "spatial sequences" of Fluo-4 and warped Fura Red imaging data. A single reservoir projects these spatio-temporal sequences into a higher-dimensional space, and a subsequent dimensionality reduction technique clusters all gray-level pixels into background and object sets.

In this study, we tested principle component analysis (PCA), extreme learning machine (ELM)-based autoencoder and the generalized Hebbian learning (GHL) rule to calculate the unsupervised readout weights $(\mathbf{W}^*)^{out}$ (shown by dashed lines in Figure S1).

Adopting an orthogonal base transformation, PCA projects the reservoir states into a linearly uncorrelated low-dimensional space by maximizing the following optimization problem:

$$(\mathbf{W}^*)^{out} = \underset{\text{s.t.}\ (\mathbf{W}^{out})^T\mathbf{W}^{out} = \mathbf{1}_{N_y}}{\operatorname{argmax}} \ ||(\mathbf{W}^{out})^T\mathbf{Z}\mathbf{Z}^T\mathbf{W}^{out}||_2 \tag{S1}$$

where $\mathbf{Z}\mathbf{Z}^T$ is the covariance matrix of zero-mean extended reservoir system states and $\mathbf{1}_{N_y}$ is $N_y \times N_y$ identity matrix (N_y denotes the number of output units). The optimal $(\mathbf{W}^*)^{out}$ can be provided by eigenvectors corresponding to N_y largest eigenvalues of the covariance matrix, $\mathbf{Z}\mathbf{Z}^T$.

In the case of single output neuron, the generalized Hebbian learning (GHL) rule offers an alternative with lower learning cost to calculate the largest eigenvalue [1]. Following this learning rule, the connection strength between the reservoir neurons and the output is recursively updated

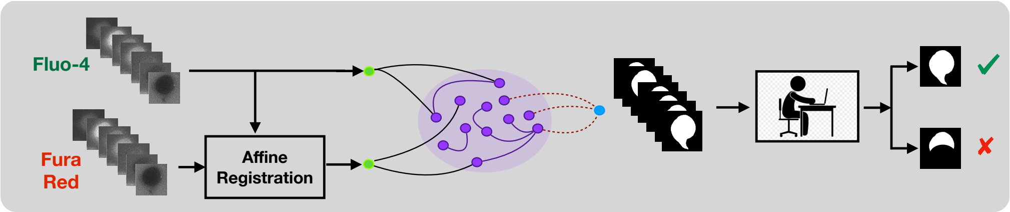

Fig. S1. Semi-manual pixel-wise ground-truth generation system for Task 1. [Graphics created with MATLAB and the Image Processing Toolbox Release 2019b, The MathWorks, Inc. (mathworks.com/products/image.html)]

to find the optimal values:

$$\Delta \mathbf{W}^{out}(t) = \eta(\mathbf{y}^*(t)\mathbf{Z}^T(t) - \mathrm{LT}[\mathbf{y}^*(t)(\mathbf{y}^*)^T(t)]\mathbf{W}^{out}(t)) \tag{S2}$$

where $\mathbf{y}^*(t) = \mathbf{W}^{out}(t)\mathbf{Z}(t)$ is a linear output neuron, η denotes the learning rate and $LT[.]$ sets all matrix elements above the diagonal equal to 0.

In (ELM)-based autoencoder [2], a low-dimensional, random representation of the reservoir states, H, is obtained by using random weights, $(\mathbf{W}^{out})^0$, presented to a non-linear activation function, $g(.)$:

$$\mathbf{H} = g((\mathbf{W}^{out})^0\mathbf{Z} + \mathbf{b}^0) \tag{S3}$$

where $\mathbf{b}^0 \in R^{N_x \times 1}$ is the bias. Then the dimension reduction mapping is calculated by solving the following regression problem with regularization parameter, λ:

$$(\mathbf{W}^*)^{out} = \underset{\mathbf{W^{out}}}{\mathrm{argmax}} \quad ||\mathbf{W}^{out}\mathbf{H} - \mathbf{Z}||_2 + \lambda||\mathbf{W^{out}}||_2. \tag{S4}$$

Having the $(\mathbf{W}^*)^{out}$ as well as the extended state matrix, $\mathbf{Z}$, unsupervised output is calculated by applying Eq. 2 of the main manuscript.

Pixel-wise continuous values returned in the output are subsequently converted to binary values (i.e., $\{0, 1\}$ for {background, object}) via thresholding. The predictions are then visually evaluated and classified as "good" or "bad", reducing the laborious pixel-wise labeling to a binary classification task.

To be used in our Jurkat T-cell segmentation pipeline (Figure 2C of the main manuscript), we manually defined 80 rectangular (128×128 pixel) regions of interest (ROIs) in image sequences obtained from 10 recording experiments. Consequently, 40 Jurkat T-cells with various morphological properties were elicited from both Fluo-4 and Fura Red imaging data. For each cell, 7000 available frames were temporally uniformly downsampled to present the reservoir with 700 frames per emission. The states of reservoir units were afterward harvested over $700 \times 128 \times 128$ iterations. Customized to a single cell data sequence, readout weights for an output unit with sigmoid activation function were then estimated by PCA, ELM-based autoencoder, and the generalized Hebbian learning rule. Given the model of Eq. 1 of the main manuscript, the input and recurrent connection matrices $\mathbf{W}^{in}$ and $\mathbf{W}$ were generated randomly according to adjustable parameters such as scaling of $\mathbf{W}^{in}$, the reservoir size, N_x, sparsity, and the spectral radius of $\mathbf{W}$. In this study, N_x was set to 50, and each reservoir node was randomly connected to 10 of the other nodes. For each cell, the learning parameters were optimized through extensive grid search. After comprehensive visual inspection, it was clear that among dimension reduction methods, the generalized Hebbian learning rule returned the highest number of well-labeled frames (93% of all the available frames, compared to 72% and 86% for the PCA and ELM methods). Thus, in the main manuscript, we report results based on the reference set provided by the GHL method. Figure S2 illustrates 4 samples of frames marked as successfully labeled. Further, we employed this semi-manual pixel annotation method to generate ground truth segmentation for a separate set of 22 primary T-cells (50 frames each with 128×128 pixel ROI size) elicited from 5 recording experiments. This additional set of annotated cells was used to assess generalizability capabilities of RC-based cell segmentation models (introduced in section 2.4.1 of the main text).

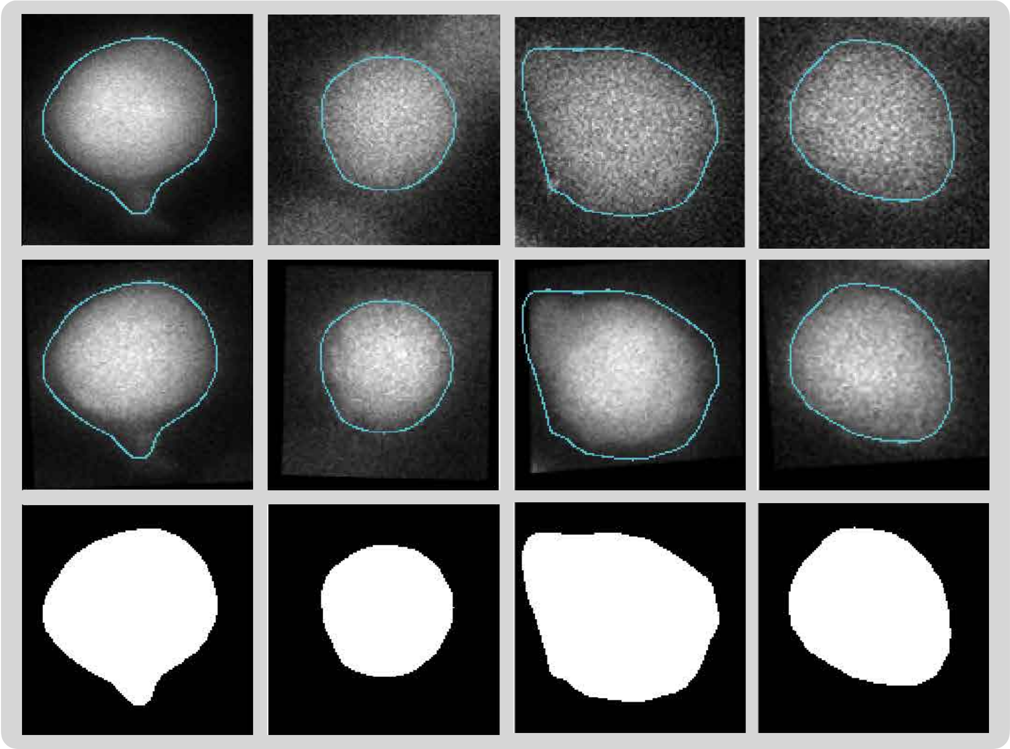

Fig. S2. Results of unsupervised pixel clustering in four Jurkat T-cells. Top: a frame (128 × 128 pixels) of the Fluo-4 Ca^{2+} imaging sequence. Middle: corresponding Fura Red imaging data. Bottom: the generated ground-truth mask. Blue contours indicate the mask boundaries. [Graphics created with MATLAB and the Image Processing Toolbox Release 2019b, The MathWorks, Inc. (mathworks.com/products/image.html)]

2. SUPPLEMENTARY NOTE 2: SEMI-MANUAL PIXEL-WISE DATA ANNOTATION METHOD IN TASKS 2 AND 3

To semi-manually create the ternary mask (classes: background, T-cell, bead), we again relied on the ability of RC in time-series prediction. Specific to each Fluo-4 imaging sequence, we assigned local RCs (with nine inputs and single output neurons) to each window of 3×3 neighboring pixels (see Figure S3). Each RC, locally, utilizes the unsupervised generalized Hebbian learning rule to return a binary value for foreground and background pixels over time. The foreground objects are later marked as cells or beads based on a user intervention where the objects and their corresponding bounding boxes are labeled on the average image acquired over the whole frames. Through a human assessment, the consecutively well-labeled frames are then collected to create the ground truth set for tasks 2 and 3 detailed in the main manuscript. Figure S4 illustrates two samples rated as well-labelled labeled frames.

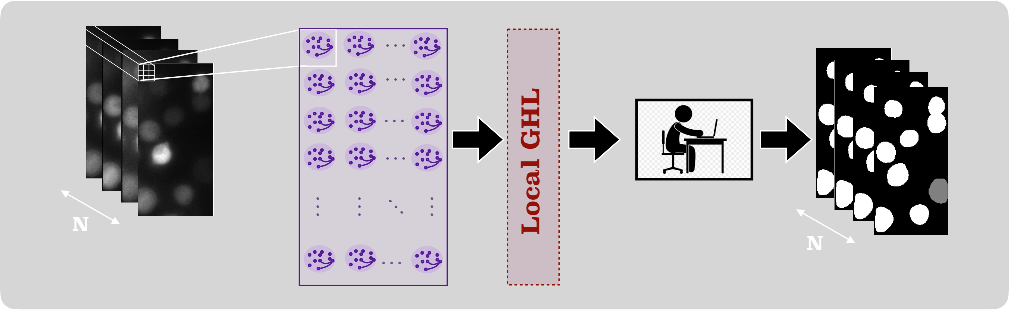

Fig. S3. Semi-manual pixel-wise ground-truth generation system for Tasks 2 and 3. [Graphics created with MATLAB and the Image Processing Toolbox Release 2019b, The MathWorks, Inc. (mathworks.com/products/image.html)]

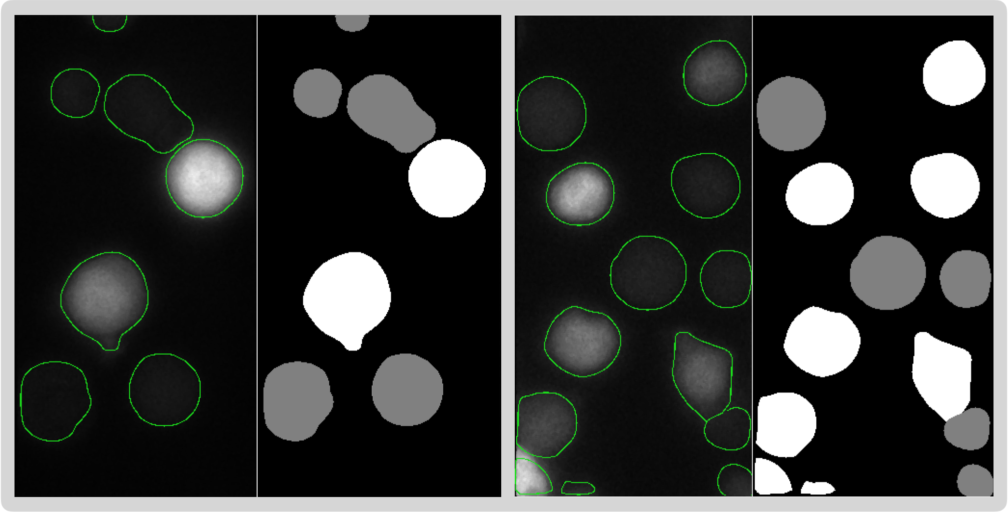

Fig. S4. Results of unsupervised pixel annotation in two frames from different imaging sequences. Left to the white line in each panel: a frame (500×250 pixels) of the Fluo-4 Ca^{2+} imaging sequence. Right: the generated ground-truth mask where gray color denotes the antibody-coated beads and Jurkat T-cells are displayed in white. Green contours indicate the mask boundaries. [Graphics created with MATLAB and the Image Processing Toolbox Release 2019b, The MathWorks, Inc. (mathworks.com/products/image.htm)]

3. SUPPLEMENTARY FIGURE 1: TEMPORAL CONTOURS EVOLUTION FOR STANDARD U-NET AND U-NET-LSTM PREDICTIONS

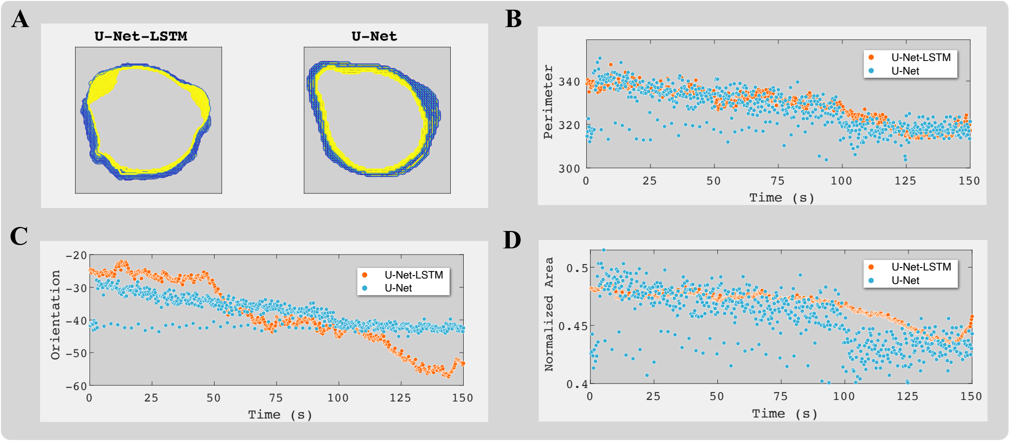

Fig. S5. Predicted temporal contours evolution for a representative T-cell and segmentation in the Fluo-4 emission data. **A:** Unlike the discrete contour trajectory returned by the U-Net, the contours predicted by the U-Net-LSTM method evolve continuously. **B:** Frame-to-frame changes in contour length of the predicted masks. **C:** Mask orientation over time. **D:** Mask normalized area over time. [Graphics created with MATLAB and the Image Processing Toolbox Release 2019b, The MathWorks, Inc. (mathworks.com/products/image.html)]

4. SUPPLEMENTARY FIGURE 2: CONFUSION MATRICES CORRESPONDING TO THE TASK 3

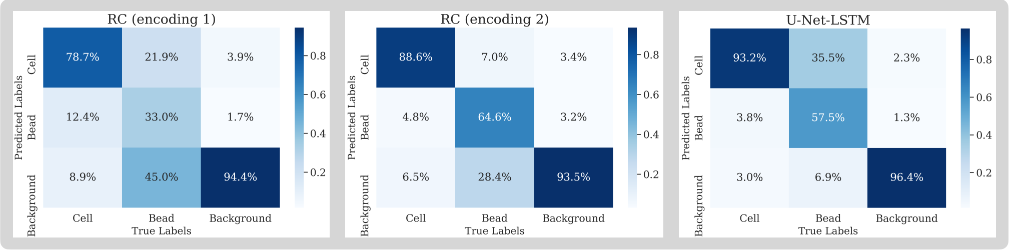

Fig. S6. Class-wise normalized confusion matrices corresponding to the performance evaluation of reservoir computing models and the U-Net-based LSTM for Jurkat T-cell and bead segmentation in single-emission recordings (**task 3**; test set: 1155 frames from 5 imaging sequences, frame size: 500 × 250). Class-wise accuracy and Dice coefficient metrics are summarized in Table 4 (main text). [Graphics created in Python 3.0, seaborn: statistical data visualization, ver. 0.11.1 (seaborn.pydata.org)]

5. SUPPLEMENTARY VIDEO 1: TEMPORAL CONTOURS EVOLUTION FOR RESERVOIR COMPUTING MODEL AND STANDARD U-NET PREDICTIONS

Visualization of predicted contours evolution for a representative T-cell and segmentation in the Fluo-4 emission data shown in Figure 6.

6. SUPPLEMENTARY VIDEO 2: TEMPORAL JURKAT T-CELLS AND BEAD SEGMENTATION

Visualization of a representative stream of Ca^{2+} Fluo-4 emission imaging data, reference segmentation, and predicted labels computed by the proposed RC model with encoding scheme 2. In the ground truth frames, gray color denotes the antibody-coated beads and Jurkat T-cells are displayed in white.

REFERENCES

1. T. Sanger, "Optimal unsupervised learning in a single-layer linear feedforward neural network," Neural networks **2**, 459–473 (1989).
2. E. Cambria *et al.*, "Extreme learning machines [trends & controversies]," IEEE intelligent systems **28**, 30–59 (2013).
